# Experiences of loneliness among older deaf adults who use sign language

**DOI:** 10.1093/jdsade/enaf070

**Published:** 2025-11-18

**Authors:** Anu H Jansson, Maria Kursi, Kaisu H Pitkälä, Timo Strandberg, Laura Rautiainen, Tarja Ylimaa

**Affiliations:** Department of General Practice and Helsinki University Hospital, Unit of Primary Health Care, University of Helsinki, Helsinki, Finland; Finnish Folk High School for the Deaf, Helsinki, Finland; Department of General Practice and Helsinki University Hospital, Unit of Primary Health Care, University of Helsinki, Helsinki, Finland; Helsinki University Hospital, University of Helsinki, Helsinki, Finland; Center for Life Course Health Research, University of Oulu, Oulu, Finland; Department of General Practice and Helsinki University Hospital, Unit of Primary Health Care, University of Helsinki, Helsinki, Finland; The Finnish Association for the Welfare of Older Adults, Helsinki, Finland; The Finnish Association for the Welfare of Older Adults, Helsinki, Finland

## Abstract

Loneliness is recognized worldwide as a risk to well-being and health. It emerges from the discrepancy between our expectations of social relationships and the reality. However, very little is known about the experiences of loneliness among older deaf adults. The aim of the study was to describe the experiences and expressions of loneliness among 12 older deaf adults who use sign language. We interviewed them, and the interview transcripts were analyzed inductively. Loneliness manifested itself in a variety of ways in the narratives, facial expressions, postures, movements, gestures, and the very individual-specific signing of the respondents. The narratives illustrated the loneliness they experienced and how they coped with it. To our knowledge, this is one of the first studies on loneliness among older deaf adults who use sign language. It should be acknowledged: Discovering and understanding this hidden phenomenon is the key to alleviating it. Future studies should explore tailored interventions and inclusive communication strategies to alleviate loneliness. Involving older deaf adults who use sign language in cocreating solutions will be essential.

## Introduction

Enhanced social connections play a crucial role in boosting overall life satisfaction (Antonucci et al., 2017). The feeling of being understood by others is extremely important for the vast majority of people (Holt-Lunstad, 2022). Being able to speak the same language with peers, to enjoy good communication with others, and to share and reflect on mutual experiences are key factors in social participation, loneliness prevention, and loneliness alleviation among older adults (Cheung & Zhang, 2023; Jansson, 2020).

This is significant, as loneliness and social isolation are associated with various adverse health outcomes, such as morbidity and dementia, poor health-related quality of life, reduced psychological well-being, and increased mortality risk (Cacioppo et al., 2014; d’Hombres et al., 2024; Holt-Lunstad et al., 2010; Holt-Lunstad et al., 2015; Jansson et al., 2017; Rautiainen et al., 2024; Shen et al., 2022). Furthermore, social isolation among older adults also causes substantial increases in annual healthcare spending (Shaw et al., 2017). In Finland, communication has been—and unfortunately still is—significantly harder for older deaf adults who use sign language than for the hearing population (Katsui et al., 2024; Koivisto & Katsui, 2024).

Older adults in this paper refer to individuals aged 65 and over. This age group of people who use sign language in Finland is part of a linguistic and cultural minority. It is numerically small, comprising approximately 1,000–1,500 individuals (Torboli & Pulkkinen, 2013). It comprises several language groups in addition to users of Finnish Sign Language, such as users of Swedish and Russian Sign Language, as well as immigrants with their own native sign languages (Torboli & Pulkkinen, 2013). According to a survey of this population group (*n* = 421) conducted by the Finnish Association of the Deaf in 2012, 36% of the respondents felt lonely at least some of the time (Torboli & Pulkkinen, 2013). For comparison, in the Helsinki Aging Study (2019–2022), 28% of home-dwelling older adults aged 75 and over (*n* = 989) reported experiencing loneliness (Rautiainen et al., 2024), and, in a separate study among older adults living in assisted facilities (*n* = 854), 55% reported suffering from loneliness (Kolster et al., 2025).

Many of the older deaf adults in the survey of Torboli and Pulkkinen (2013) were born into hearing families and were provided no opportunities to learn or acquire language naturally in their early childhood. Oralism, a teaching method that prohibited the use of sign language and emphasized learning to speak, was prevalent in schools for deaf people at the time. Sign language was not considered equal to other languages, nor as enabling natural interaction. In fact, oralism led many older deaf people to internalize the negative belief that sign language was wrong and something to be embarrassed about (Katsui et al., 2024; Koivisto & Katsui, 2024). Life-long experiences such as these are known to exacerbate feelings of loneliness, to which both childhood and life-course experiences are related (Tiilikainen et al., 2024).

Beyond the sign language community and excluding interpreters, few nondeaf people nowadays know how to sign in Finland. Older deaf adults who use sign language in Finland are limited in terms of accessing everyday interactions and services in their own language. Those living in sparsely populated areas may have no peers at all. Interpretation services are essential and are often a prerequisite for mutual understanding. However, many feel that their everyday interaction and ability to communicate are overly dependent on such services: “Tomorrow you’ll understand for four hours, the day after tomorrow for three” (Laakkonen, 2019). This quote, drawn from an interview with a Finnish deaf person, illustrates the unpredictable nature of communication access. It highlights how reliance on interpretation services can restrict autonomy and contribute to feelings of exclusion, especially when opportunities for interaction are rationed or inconsistent. A total of 3,602 people of all ages in Finland are entitled to interpretation services (Kelasto, 2022).

Communication problems among this group of people worsened during the COVID-19 crisis for various reasons, including the need to wear face masks, physical and social distancing, and a lack of relevant information. For example, face masks hindered lip-reading and facial expression, which are crucial for effective communication. Physical and social distancing measures further isolated deaf individuals, making it difficult to engage in social interaction and to receive support (Mansutti et al., 2023; Naylor et al., 2020). These challenges widened the gap between expectations and reality in social situations, leading to stronger feelings of isolation and disconnection from society. In a Finnish longitudinal study examining the effects of the COVID-19 pandemic, the prevalence of loneliness among older home-dwelling adults increased from 26% to 30% between 2019 and 2021 (Knuutila et al., 2023).

The discrepancies between social needs, expectations, and reality further fuel loneliness, as it is precisely these discrepancies that give rise to experiences of loneliness (Perlman & Peplau, 1982). People experience this gap in their own unique way (Victor et al., 2009, 43) and regardless of whether or not there are people around (Jansson et al., 2023). Their experiences may also be related to other concepts such as experiencing invisibility, alienation, and social death (Borgstrom, 2017; Jansson, 2020; Jansson et al., 2023) that offer a more nuanced theoretical lens through which these experiences can be understood. They help illuminate how the loss of recognition and connectedness may shape the emotional and social realities of deaf older adults.

Recent research has shown that stigma and self-stigma related to hearing loss can further intensify feelings of loneliness and social isolation among older adults (da Silva et al., 2023; David & Werner, 2016; David et al., 2018; Shukla et al., 2020). Stigma may arise through social experiences, cultural perceptions, and personal beliefs, and it can negatively affect identity and social interaction, as well as the willingness to use assistive devices such as hearing aids (David & Werner, 2016; David et al., 2018). These findings are also relevant in the context of older deaf adults who use sign language, whose experiences of language use, exclusion, and limited access to communication may be compounded by internalized stigma and societal attitudes.

The Finnish Association for the Welfare of Older Adults, in co-operation with the Finnish Association of the Deaf, piloted the Circle of Friends group intervention among older deaf adults who use sign language in 2019–2021, with the aim of addressing loneliness in this underserved population. One of the authors (TY) coordinated the Circle of Friends pilot. TY’s insights are based on their direct involvement in the planning and implementation of the groups. A more detailed description of the process has been published elsewhere (Kursi et al., 2022). The Circle of Friends was chosen for the pilot because of its effectiveness. A randomized controlled trial two decades ago demonstrated improvements in older participants’ health, cognition, and overall well-being, along with reductions in healthcare usage and mortality (Pitkälä et al., 2009).

Long-term follow-up revealed that 90% of the Circle of Friends group participants felt that their loneliness had been alleviated, and 67% continued independent meetings after the facilitated 3-month period (Jansson et al., 2018). Later, the Circle of Friends was acknowledged by the European Commission as one of the best interventions addressing loneliness among older adults in the field of mental health in the European best practices portal (European Commission, 2024). This recognition is based on the intervention’s proven effectiveness, scientific validation, and broad societal impact (European Commission, 2024). Those participating in the intervention meet their peers in a closed group 12 times over 3 months, with two trained facilitators. The participants discuss loneliness and coping mechanisms related to it with the help of the facilitators, and they form friendships. Many of them enjoy long-term peer support after the 3-month facilitated period (Jansson & Pitkälä, 2021).

However, older deaf adults who use sign language and who participated in the Circle of Friends pilot intervention for deaf found loneliness and its related concepts rather difficult to explain. Some were unable to find signs to express themselves and their experiences of loneliness directly. They did so indirectly, using many euphemisms. They found it hard to produce and understand Finnish signs related to the theme of loneliness. Expressions such as “feeling lonely when surrounded by other people” seemed difficult in sign language, for example. Furthermore, for some, the group discussion was the first time in their lives that they had been offered the opportunity to express their experiences of loneliness. They seemed to feel confused in this new group situation, despite already having conveyed their experiences of loneliness in the individual interviews before the group started.

Our theoretical background on loneliness, combined with these empirical observations, provided us with a clear justification to investigate loneliness among older deaf adults. At the same time, this initiative addressed a significant research gap in this area. The unique experience of loneliness is both valuable and challenging as an object of study. Emphasizing personal experiences may also contribute to commonly accepted knowledge, enhancing the common understanding of loneliness (Brownie & Horstmanshof, 2011; Jansson, 2020; Tiilikainen & Seppänen, 2017). However, experiencing loneliness to others may be difficult (Victor et al., 2005). Yet, it is necessary to share one’s narrative because others may not recognize an individual’s loneliness (Jansson & Strandberg, 2022). Language plays a key role in expressing and making loneliness tangible (Ettema et al., 2010). Therefore, in this study, we sought to highlight the experiences of loneliness among deaf older adults who use sign language as they perceive it in their own language in Finland.

## Aim and methods

### Aim

The aim of this study was to explore loneliness among deaf older adults who use sign language. Accordingly, the research question guiding this study is: How do deaf older adults who use sign language experience and express loneliness?

### Recruitment and interviews

We recruited 12 older deaf adult volunteers who use sign language in the Helsinki Metropolitan Area and sparsely populated areas, to be interviewed in the spring of 2022. Participants were recruited through outreach work conducted by the Finnish Association for the Welfare of Older Adults and the Finnish Association of the Deaf, who actively engaged with sign language communities across Finland. Information about the study was also disseminated via announcements in the Gerontologia journal (Kursi et al., 2022) and in local newspapers. In addition, regional workers from the Finnish Association of the Deaf contacted their own clients directly. Older adults who were interested in participating reached out to a researcher who used sign language (MK), which likely supported accessibility, trust, and cultural–linguistic relevance in the recruitment process.

The fact that the contact came from the potential participants ensured their voluntariness in the study. Older deaf adults who use sign language received written information about the study through a notice from the Finnish Association of the Deaf and the Finnish Association for the Welfare of Older Adults. After contact had been established, each one received an information sheet. In addition, the interviewer conducted a video call in sign language with each participant beforehand to explain the study in more detail. They signed a written consent form independently, and they could withdraw from the study at any time without it affecting their care or any other services they used.

The interviews were carried out in the summer of 2022. We explored subjective, individual expressions of loneliness among the interviewees, as well as common underlying features. The interviewer was a native signer (MK), which enabled the respondents to express their experiences and thoughts naturally in their own language, namely, sign language. MK listened attentively and respectfully to their stories, ensuring that the interviewees had the interviewer’s full attention during the interviews (Heyl, 2001) in a safe atmosphere, which was crucial for such a sensitive subject (Kirkevold et al., 2013). This contributed to a confidential and safe interview environment, which was essential. The interviewees could choose the interview setting, which was usually their own private home or its immediate surroundings.

The interviews were video recorded. This enabled the interviewer to focus fully on communicating in sign language. Signing is a visual language that uses facial expressions and body language (Sutton-Spence & Woll, 1999). Eye contact is an essential part of the interaction. It was therefore important not to break eye contact by, for example, taking notes. The interviewer used a smartphone on a tripod, which had the necessary recording features. The seating or standing arrangements enabled natural and direct eye contact during the conversation.

The participants were interviewed either individually (*n* = 7) or in a focus group (*n* = 5). The duration of the interviews, which included an orientation phase, ranged from 60 to 90 min, with an average length of 65 min. Given the information from the Circle of Friends group, as well as the interviewer’s long professional career working with older deaf adults who use sign language, we realized that the participants might not be used to sharing their thoughts. We also assumed that they might find it difficult to express their wishes and feelings as well as their experiences of loneliness. We therefore used techniques that facilitated discussion. As part of the orientation phase, before each interview, the interviewer engaged in an informal conversation with the interviewee to observe how they signed about various topics and which signs they used. This paved the way for a commonly understood sign language during the interviews. The interviewer used plain language expressions and terms. If the interviewee was not able to start their narrative on some of the more difficult themes, the interviewer provided a few response options if necessary. The interviewees were free to describe their experiences in their own ways.

In each interview, there was a brief discussion about the difference between being alone and experiencing loneliness. To clarify the concepts, the interviewer used different signs for these terms [in sign language]:

Let’s discuss the difference between loneliness and being alone.

Loneliness is almost always a negative experience. Loneliness is not a choice; people end up lonely for various reasons. Lonely individuals tend to expect more from their social relationships than they receive.

Being alone by choice, or solitude, is different from suffering from loneliness. Being alone may well be a voluntarily chosen state and may be positive and needed.

The experts who planned the interviews (AJ, MK, TY) jointly created a list of thematic questions to guide their progress (see Appendix 1). However, the main aim was to proceed on the interviewees’ own terms so that they felt safe and had a genuine opportunity to share their experiences in their own way. The process was flexible according to the individual signs used by the interviewee, and no two people were asked the same questions in the same way.

The interviewees knew in advance why the interviewer was coming and what topic was to be discussed, which gave them time to prepare and to think about the theme. As a result, two interviewees started talking about their experiences in the hallway as soon as the interviewer arrived—or was leaving. Some found it difficult to discuss loneliness, whereas others were slightly apprehensive about the interview setting and only really started talking about their experiences when the video camera was turned off. In these situations, they talked about their childhoods, their experiences of ill treatment, and their current life circumstances related to loneliness. The interviewer made notes in the form of a field diary immediately afterward, sitting in their car.

### Transcription

There were a number of hours of video data, of which 280 min were relevant to the research question. In addition, 10 pages of field notes were used to complement the video material. The interviews were transcribed into written Finnish prior to analysis. This decision was made to enable the full participation of the multidisciplinary research team, which included some of the most experienced and internationally respected scholars in the field of loneliness research among older adults. While these experts brought decades of experience in both qualitative and quantitative research, they did not know Finnish sign language. Transcription into written Finnish was therefore necessary to ensure that their expertise could be fully integrated into the analysis process. To support inclusive collaboration and maintain the integrity of the original narratives, one or two professional Finnish sign language interpreters were present at all research group meetings, facilitating communication between signing and nonsigning members.

Transcription was a laborious task, as sign language has its own grammar. Furthermore, the videos showed the facial expressions, body language, and forms of expression that are linked to the culture of the Finnish sign language community. The interviewer and transcriber (MK) found that simply transcribing from Finnish sign language into written Finnish did not convey emotions, most of which were expressed through facial expressions and gestures. Therefore, neither direct translation nor mere transcription from sign language into Finnish worked for the purpose of this study. The authors (AJ, MK, and TY) decided to include descriptions of body language and gestures in brackets both in the transcript and when reporting, if they added value. The aim of this was to present the results in a transparent manner.

For example:

I have no friends even though I wish I did. So I have to walk to the shop alone, and after I’ve finished shopping I walk back with my Nordic walking poles [the interviewee signed this with an expression highlighting silence and isolation from others].

### Thematic analysis

The thematic analysis was conducted collaboratively by a multidisciplinary research team. The team included researchers with extensive expertise in loneliness among older adults, both in Finland and internationally. We carried out the thematic analysis (Naeem et al., 2023) following the principles outlined by Braun et al. (2019), as presented in the Handbook of Research Methods in Health Social Sciences. This approach emphasizes a flexible and interpretive process of theme development, in which the researcher actively scrutinizes the data while remaining open to what it may reveal or suggest. Initially, we skimmed through the transcribed text quite quickly, allowing the themes and entities that connected the excerpts to emerge. We then read through these texts and compared them with the videos.

The coding was carried out manually without the use of qualitative data analysis software, allowing the researcher to remain intimately involved in the interpretive process. Each theme in the transcribed text was given its own color or code. Codes were initially created based on recurring expressions, gestures, and signs that appeared across interviews. For example, expressions of loneliness were coded not only through verbal content but also through repeated nonverbal cues such as downward gaze, pauses, or specific facial expressions. The themes began to emerge during the researchers’ discussions and, as they combined the codes, gradually became clearer and more coherent. This process followed an inductive approach, where themes were not predefined but emerged from the data itself. Meanwhile, we felt that some of the themes covering the life circumstances of older adults in Finland were too recognizable in the small sign language community. We therefore excluded all factors related to the risk of identification to ensure anonymity. When we wrote up the results, we checked the responses against the signs, facial expressions, and gestures shown in the videos to ensure that they reflected what the interviewee had intended.

## Ethical considerations

We adhered to strict ethical principles in our study, ensuring respect for the dignity and autonomy of the participants. Formal Institutional Review Board approval was not used, nor was it required, as the study did not involve any of the conditions outlined by the Finnish National Board on Research Integrity (Finnish National Board on Research Integrity TENK, 2019) that would necessitate ethical review. Participants self-identified as capable of participating in the study. This assessment was confirmed by either a regional sign language worker familiar with the community or an interviewer with extensive experience working among deaf older adults who use sign language. The participants managed their daily activities independently, with the exception of one who lived in an assisted living facility but was capable of making their own decisions without daily assistance. Ample time was provided for signing the consent form to accommodate the needs of these potential interviewees. They signed a written consent form independently, and they could withdraw from the study at any time without it affecting their care or any other services they used. They could also forbid the use of their interview data at any stage, either during the process or afterward. We considered the participants’ sign language and related needs carefully during the interviews and data analysis, and explained the topics related to the themes in some detail. Furthermore, we compared the transcribed text to the videos to ensure that the results reflected the participants’ narratives accurately.

## Results

### Participants

The interviewees were from various parts of Finland, aged between 65 and 81. We do not report their gender identity to maintain anonymity. Eleven of them lived at home in an apartment building or a private house, and one lived in an assisted living facility. Seven of them lived alone. Most of the interviewees had children or other relatives with whom they kept in touch. All of them had been born into hearing families, and most had attended a school for the deaf.

### Loneliness was both felt and expressed through signs

Many interviewees described loneliness in concrete, descriptive terms through their everyday activities. Their facial expressions and body language revealed the tones in which they described their experiences and gave the experience of loneliness its true meaning.

I miss something [looks down with a sad facial expression and touches their chest with both hands, almost closed and then their hands move up to their face into a crying sign]… Hard to be alone… I try to bear it… I should do something, but I just can’t. I just rest.

Many described their experiences in vivid and non-verbal terms, and their experiences and emotional states were conveyed through facial expressions, body language, and narration.

In loneliness… I’m tired. I’m tired. I do feel tired… I must go to the swimming pool every now and then. Swimming helps a lot. I dive under the water [sad expression]. The cold helps and refreshes me. I swim and dive again, it helps [relaxed expression communicates relief and invigoration].

### Subjective experiences in the face of global disruption

The COVID-19 pandemic began in the spring of 2020, and the virus also affected the daily lives of the interviewees. They were asked how they had felt about the pandemic. They described their experiences and feelings in a wide range of ways, referring to the use of masks and social distancing in particular.

I always had to keep a distance from others [the interviewee signs this animatedly and descriptively with a sceptical expression]. It was awful, you could say a bit distressing, really [shakes head vigorously]. It was awful [places a hand on their heart].

I feel like before COVID I had a lot of friends. But now they’ve started to disappear. There’s nothing I can do. I have to accept it [sighs].

The Russian war of aggression against Ukraine, which began in the spring of 2022, also caused feelings of insecurity, uncertainty, and ill-being among the interviewees. They said they were left alone with their fears and worries. The only source of information and support available to the participant was when sign language news about the war was broadcast on TV. In the absence of desired social contacts or support, these experiences contributed to feelings of loneliness. They wanted sign language conversation and peers with whom to share their thoughts and concerns in their own language, and with whom they could process the information they had received.

I kept watching the sign language news on TV and checking to see what time the next news broadcast was! I watched, I watched, and I watched [looks anxious and fearful]. Then when I went to bed, I stared at the ceiling with my head spinning, full of thoughts.

### Loneliness in time and place

Loneliness was also described as being connected with time and life-course situations, such as retirement.

[pensive expression] I worked eight hours a day for a long time and then I retired. At first it made me sad when my spouse went to work or to run an errand for a few hours. I felt sort of helpless and I got this feeling of longing. Then when my spouse returned home, I felt relieved to be able to talk to them. Is this loneliness, I don't know… Then when they were home again, I was happy to be able to talk to them.

Loneliness was also described as a place-specific experience for which the living environment was significant. For example, one respondent recalled being lonely in their small studio apartment.

I: So, you said, you don't feel lonely when you're out and about in the countryside?

R: No, I don’t.

I: But you feel lonely when you’re in your apartment?

R: Yes.

I: Why do you feel lonely in your apartment?

R: I find it hard to go out, Nordic walking, for instance. I don't really understand why. It's just hard. Last night I forced myself to go, despite my aches and pains. In the countryside I can move freely without any worries. Someone comes to say hello every now and then. When I'm in my apartment, I just get agitated [irritated expression].

### Loneliness among peers

Loneliness was intertwined with social relationships and a longing for them. The respondents described it as lacking a friend, a close one, a close relationship, or meaningful interaction. “I have no friends. No-one to chat with. Also, I’d like to go out with a friend [interested expression], but I have no friends” [dejected expression, mouth turned slightly downward].

The interviewees found participating in events in sign language meaningful. They reported feeling a sense of belonging. They perceived meetings with peers as boosting their mood. “After attending the senior club, I think at home about how nice it was and recall the events in my mind. I really feel good. I’m happy, calm, and in a good mood.”

However, various sign language gatherings in which interviewees had felt excluded were described as difficult. Such situations caused disappointment and made them feel like outsiders. “In the group they focused on signalling to others and ignored me. He raised his hands and looked away.”

The other deaf people knew each other well and often signed among themselves. They didn't bother with me… I was hoping they would ask me to join the conversation, but they didn't. Only one asked… Then we went home.

Some interviewees described the sign language community as hierarchical, dividing people into “stupid” and “clever.” Many respondents felt inferior in comparison to other “cleverer” people.

Sometimes it was nice companionship, I was included. But little by little, I wasn’t given a turn to speak, while the others in the group belittled me as stupid and didn't bother to listen. They only talked to the clever ones. They had lively discussions because they had so much knowledge. I had no chance to speak. I sat there saying nothing.

### Navigating loneliness in hearing-dominant contexts

The language barrier with nonsigners, mainly the hearing, also seemed to form a barrier to social interaction and connecting with other people. On the one hand, the respondents described this kind of loneliness as typical and acceptable, as if it were the norm, but on the other hand they perceived it as uncomfortable and as reinforcing the feeling of loneliness. Many of them described the stress related to being with and spending time among hearing people as a signer.

Yes, I always feel lonely during leisure-time activities with hearing people. Whenever we travel to a different place, I sit alone while other people talk to each other. In the evening, I see my spouse, and it's nice to chat in sign language. I see others having a good time, but I'm lonely.

The interviewees often felt excluded if they had no sign language interpreter during interactive and information-sharing situations. One even expressed the wish that a close friend would call in such situations. “When I’m lonely among the hearing, I look at my phone and wait for it to ring. At least then I could chat with someone!”

One interviewee felt that the needs of older deaf adults who use sign language were not heard or listened to enough. These experiences were related to employees working with older adults who lacked sign language skills, and to assisted living facilities where no signing was used at all. “I think the hearing should understand the deaf. Sign language brings good spirits, well-being, comfort, and enjoyment of life. Honesty too.”

### Technology as an opportunity and a challenge for social interaction

The respondents used a variety of digital means of communication. However, the tools did not meet all their expectations in terms of social relationships.

Living here at home, I do feel lonely, even though I’m in touch with other deaf people. We chat on Messenger, Skype, and WhatsApp, for example. But I still feel lonely… I'd like some physical conversation – there's no senior club around here [expression of disbelief, disappointed astonishment]!

Some interviewees said that technology offered many opportunities to deaf people, opening up the world and enabling better access to information and contact with others, including hearing people.

For example, 30–40 years ago I was quite lonely in the family, with only my mother to talk to. Nowadays everything is better because we have smartphones and things like that. In the past, there were no computers or televisions. Today there are, and I get information through them.

One interviewee said that they had found a way to prepare for video calls and considered this a major success.

I showed my list to the recipient of the call so that he saw I had things to say. I started signing things off the list. Having said all the things I wanted to, one by one, I told my peer that it was their turn. I reminded them before the call to also write down on paper what they wanted to say.

Some interviewees stated that remote meetings also had their drawbacks. They felt that there were more misunderstandings during video calls than in face-to-face meetings. This affected the motivation to use technology for communication. Others said that “normal face-to-face” sign language conversation was more natural: In nature, for example, it is easy to pick up topics from the environment. “I’m not used to looking at a [signs ‘small’] screen when chatting with friends. I’m not used to it.”

It would be more relaxing to have a coffee, walk to another place, and sit down to watch passers-by while chatting with someone. That would be nicer than being at home in front of a screen [shakes head vigorously].

## Discussion

This study describes and highlights the varied and rich experiences of loneliness, as well as lonely situations in everyday life faced by Finnish older deaf adults who use sign language. It was conducted across Finland, in the Helsinki Metropolitan Area and in sparsely populated areas. Characteristic loneliness manifested itself in many ways in the respondents’ facial expressions, postures, movements, gestures, and very individual signing. In line with previous studies (Newall & Menec, 2019), loneliness was experienced when alone and when surrounded by others, including both hearing people and deaf sign language users. Experiences related to the COVID-19 pandemic, the Russian war of aggression against Ukraine, and technological dominance with regard to communication were prominent in their responses. Like Karisto and Tiilikainen (2017) also have noticed, interviewees found it difficult to discuss loneliness, or brought up issues only after the interview had concluded.

Reflecting on the study findings, we suggest that it is worth considering how the stigma associated with sign language might compound the experience of loneliness (da Silva et al., 2023; David & Werner, 2016; David et al., 2018; Shukla et al., 2020). Internalized shame about using sign language, a result of historical oralism and societal attitudes (Katsui et al., 2024), could exacerbate experiences of loneliness. The intertwining of these factors can be at least confusing and feel stigmatizing (Victor et al., 2005). When loneliness combines with the stigma of using sign language, it can constitute a double burden for deaf older adults. Furthermore, ageism, which unfortunately still exists, adds another layer of stigma. This could aggravate the shame and stigma experienced by deaf older adults, making their experience of loneliness even more profound.

The COVID-19 pandemic exacerbated experiences of loneliness among our participants. The specific challenges inherent in using face masks and social distancing, together with a lack of information, may have widened the gap between expectations and reality in social situations (Mansutti et al., 2023), leading to loneliness. Many older deaf adults who use sign language rely heavily on visual cues such as lip reading and facial expressions for effective communication. The widespread use of face masks significantly hindered their ability to understand hearing people. Social distancing measures further limited their opportunities for in-person interaction, which they longed for, and which is crucial for maintaining social connections. These findings are supported by previous research showing that the COVID-19 pandemic affected people with hearing loss in various ways (Mansutti et al., 2023; Naylor et al., 2020). The impact of COVID-19 on older adults’ loneliness was also observed in a Finnish longitudinal study, which found that loneliness increased among older home-dwelling adults during the pandemic (Knuutila et al., 2023). Together, these findings highlight how the intersection of age-related and communication-related vulnerabilities intensified the impact of the pandemic on older deaf adults. They also underscore the importance of inclusive crisis communication and support strategies that address both sensory and social needs.

There is a pressing need to enhance peer-to-peer services, news reporting, and interpreting services for members of the sign language community to help them cope in societal situations that could cause mental distress, or raise questions and highlight the need for information. Efforts should also be made to dismantle the language barriers that exist between older deaf adults who use sign language and those who communicate using spoken language, which affect social interactions and contribute to experiences of loneliness (Cheung & Zhang, 2023). As our findings imply, new societal contexts arising from events such as the COVID-19 pandemic and the Russian invasion of Ukraine could be detrimental to older deaf adults who use sign language, and fuel the experience of loneliness, excluded from information and support. These situations may also align with the concept of information deprivation trauma (Schild & Dalenberg, 2016), where lack of accessible and timely information leads to psychological distress and a sense of disconnection from society.

Strengthening support systems could help to bridge the gap and facilitate much-needed connection and understanding. It would also be appreciated if someone were to ask, “How are you?” in their own language. It seems that the most effective context in which to express experiences of loneliness is with one’s peers, in one’s own language. Cattan et al. (2005) refer to this as a shared social identity, which may be influential in alleviating loneliness. The significance of peer interactions and the positive effects of participating in sign language events were highlighted in the interviews.

We learned a lot while we were working on this article. Before starting our investigation, we knew, based on research findings and our extensive experience with loneliness, that experiences of loneliness could not be observed from the outside without interaction. We understood that it always had to be asked about (Jansson & Strandberg, 2022). This is still the case, but we are reconsidering the *“could not be observed from the outside”* assertion, which we would now like to re-examine in light of our research. We need to take a closer look at older deaf adults who use sign language in the hearing world. Loneliness among them is visible if one knows how to look for it. It is visible in their beautiful signs, facial expressions, and gestures. At the same time, our data suggest that loneliness is not defined solely by age. Rather, it emerges from a range of subjective and situational factors, such as communication barriers and lack of accessible information, that may affect individuals across different life stages. We hope that this perspective encourages reflection on the broader nature of loneliness and inclusion.

In the hearing environment, feelings of loneliness and of not being heard can be linked to situations in which these individuals were excluded from conversations. Becoming invisible in social situations may arouse deep feelings of loneliness and alienation. Social networks may be limited, even non-existent, especially in rural areas. Experiencing invisibility (Jansson et al., 2023) may reflect the feeling of not being recognized as persons (Pirhonen, 2017). This phenomenon is also linked to social death in some studies (Borgstrom, 2016), which occurs when people feel that they are losing their social identity and social connectedness (Jansson et al., 2023). This issue should be addressed seriously, and solutions found that make older deaf adults who use sign language visible and included in social interactions.

In addition to the challenges faced in hearing environments, the findings reveal that some deaf participants experienced exclusion within the sign language community itself. This was a particularly painful and unexpected form of loneliness, as the participants had longed for connection and belonging in spaces where their language was shared. Instead, they described feeling ignored or left out of conversations, especially when others formed tightly knit groups or engaged in lively exchanges without including them. Such experiences suggest that linguistic commonality alone does not guarantee inclusion. Social hierarchies and perceived differences in status may contribute to exclusion, even within communities that are otherwise seen as safe and familiar. Being labeled as “stupid” or not being given a turn to speak undermined participants’ sense of self-worth and reinforced their feelings of invisibility—echoing the same dynamics observed in hearing environments (Jansson, 2020). This internal exclusion within the deaf community may reflect broader issues of intragroup marginalization, where individuals are sidelined despite shared identity markers. It also deepens the understanding of loneliness as a relational and contextual phenomenon (Victor et al., 2009), shaped not only by external barriers but also by subtle social dynamics within one’s own community (Pitkälä et al., 2015).

Our findings contribute to current understanding about the multifaceted nature of loneliness and its nuances. Loneliness among older deaf adults who use sign language opens up a new path to understanding the phenomenon of loneliness, as well as exploring it further. Furthermore, when it comes to asking about loneliness, the individual’s linguistic competence and experiences with language should always be the starting point for both measurement and inquiry. The person conducting the assessment or asking the questions must understand the background, experiences, and life history of the individuals concerned (Katsui et al., 2024).

## Strengths and limitations

The study authentically highlights experiences of loneliness among older deaf adults who use sign language and is almost unique. The inclusion of descriptions of respondents’ gestures and facial expressions in the results enhances the transparency of the research. A particular strength is that the interviewer was a researcher who uses sign language as their first language. Given their extensive experience in the field, specifically in the social and healthcare sectors working with older adults, they could fully understand the issues confronting the interviewees. This strengthened the reliability of both the interviews and the reporting of the results.

However, the study also has some limitations. One is that we were unable to determine the frequency of loneliness experienced by the interviewees. They did feel lonely but could not reliably recall whether they suffered seldom or never/sometimes/often or always. Another limitation is the relatively small sample size, which was due to the laborious task of arranging the interviews and the significant resources required for the transcription phase. Looking ahead, we highlight the need to conduct more interviews on this topic in various life-course phases, for example. A third limitation is related to the process of transcribing sign language interviews into written Finnish prior to analysis, which may have influenced the interpretation of the data. Some nuances of expression, emotion, and interaction embedded in visual language may be lost or altered in written form, which is important to acknowledge when considering the depth and richness of the findings.

## Implications for policy and practice

Our findings have some notable implications for policymakers. The structural injustices and challenges faced by older deaf adults who use sign language could affect their life course, social relationships, and feelings of loneliness (Katsui et al., 2024). Given the importance of interaction, social relationships, and a sense of belonging, these issues need to be addressed. Policymakers should develop and implement policies that actively promote social inclusion. This includes creating community programmes and social activities that are accessible and welcoming, and utilizing effective models, such as Circle of Friends. Actions should also be taken to ensure that these people have consistent and reliable access to the high-quality customer-oriented interpretation services they need to be able to participate in not only various aspects of life, including healthcare, legal matters, and social services, but also a wide range of leisure activities.

Moreover, policymakers should encourage the development and use of technology that meets the specific needs of deaf older adults. This could include video relay services, captioning, and other assistive technologies that facilitate communication and reduce loneliness. Efforts should also be made to recognize and address the broader structural injustices that contribute to the marginalization of older deaf adults who use sign language. This would involve advocating for equal rights, combating discrimination, and ensuring that policies are inclusive and equitable. Focusing on these areas could help policymakers improve the quality of life among members of this population group and foster a more inclusive society.

Although digital communication tools such as Messenger, Skype, and WhatsApp offer opportunities for social interaction among older deaf adults who use sign language, they also present challenges. It would be useful to address the limitations of these tools in meeting the social needs of older deaf adults. One solution would be the tailor-made targeting of digital support at users of sign language: A peer digital mentor could provide support and guidance based on their own experiences and life course. A peer mentor could also share their own experiences and provide practical advice based on their personal life and use of digital devices and services. This would require the allocation of resources to digital support and its development to meet the needs of different minority groups.

More targeted interventions are needed to address the unique loneliness experienced by deaf older adults. New pathways should be found to open up safe and receptive spaces for discussions about loneliness among those using sign language. The significance of facial expressions, body language, and individual-specific signing in conveying loneliness in this study highlights the importance of understanding these personal narratives to develop effective interventions. Older users of sign language should be included in discussions about alleviating experiences of loneliness. In terms of empowerment, many older individuals find the keys to solutions themselves when they are encouraged, supported, and motivated (Jansson & Pitkälä, 2021). Solutions to loneliness frequently arise from the individual’s own interests, strengths, desires, and needs. Older deaf adults who use sign language should be asked for their input, rather than being offered solutions from a so-called ready-made palette.

## Conclusion

Our findings highlight the importance of recognizing the diverse ways in which loneliness manifests among older deaf adults who use sign language. This understanding could inform the development of more tailored support and effective interventions. By acknowledging the specific expressions of loneliness in this population, we could better address their needs and improve their overall well-being. Future research should continue to explore these unique expressions of loneliness and consider the cultural and linguistic contexts that shape them. Efforts should also be made to involve those concerned in the development of policies and programmes aimed at reducing loneliness, ensuring that their voices and experiences are heard and acted upon.

Addressing loneliness among this population group requires a multifaceted approach that considers their unique linguistic and cultural needs. Priority should be given to creating inclusive environments that facilitate meaningful social interactions and provide consistent access to interpretation services. Practitioners should prioritize the development of tailored interventions and support systems that facilitate the use of sign language to express experiences and connect with others. Fostering a society that values and understands diverse expressions of loneliness will enhance the well-being and quality of life among older deaf adults who use sign language, ensuring that they feel seen, heard, and supported.

## Supplementary Material

Appendix_enaf070
