# Supplementary material for: Experiences of loneliness among older deaf adults who use sign language
Source: J Deaf Stud Deaf Educ. 2025 Nov 18;31(2):335–44. doi: 10.1093/jdsade/enaf070 (PMC13324732; doi:10.1093/jdsade/enaf070)
Supplement: Appendix_enaf070 [file appendix_enaf070.docx]

**Appendix**

**Interview Protocol**

This interview protocol was used in both individual interviews and focus group discussions conducted as part of the study. The protocol was designed to explore the experiences and expressions of loneliness among deaf older adults who use sign language.

The interview began with the following orientation phase, with introductory questions:

Let’s start with a short introduction about you, what would you like to share?

What has your everyday life been like recently?

How do you usually spend your time/days?

After these initial questions, the interviewer introduced the topic of loneliness and explained the concept to the participants in accessible language. This was especially important to ensure shared understanding among older adults who use sign language. The explanation included the following:

Today we talk about loneliness.

Loneliness is almost always a negative experience. Loneliness is not a choice; people end up lonely for various reasons. Lonely individuals tend to expect more from their social relationships than they receive. Being alone by choice, or solitude, is different from suffering from loneliness. Being alone may well be a voluntarily chosen state and may be positive and needed.

The interview then continued with the following thematic questions:

What are your thoughts on loneliness, as it has been described in this interview?

What does loneliness mean to you personally?

How do you experience or understand loneliness in your own life?

How does loneliness feel to you?

In your opinion, how does loneliness appear among older deaf people in Finland?

How do you think Finnish society – including both people and public services – responds to or acknowledges the loneliness experienced by elderly deaf individuals?

What do social relationships mean to you?

Are you in contact with other people as much as you would like to be?

When you feel the need to talk to someone but no one is available, what do you usually do?

Can you tell me about your relationship with the sign language community or your local association?

What do sign language gatherings and events mean to you?

Do you always feel a sense of belonging at these gatherings?

If not, what do you think might be the reason?

Do you also participate in other communities or activities outside the sign language community?

What kinds of societal activities or services help you feel connected or supported?

In what ways do these gatherings – or other places – help reduce feelings of loneliness?

Each of these questions may lead to responses that generate follow-up questions.
